# Exploring graduate occupational and physical therapy students’ approaches to studying, self-efficacy, and positive mental health

**DOI:** 10.1186/s12909-021-02550-w

**Published:** 2021-02-23

**Authors:** Elaina DaLomba, Saji Mansur, Tore Bonsaksen, Mary Jan Greer

**Affiliations:** 1 Samuel Merritt University, 3100 Telegraph Ave, Oakland, CA 94609 USA; 2 Student Health Coordinator, Samuel Merritt University, Oakland, USA; 3 Department of Health and Nursing Sciences, Inland Norway University of Applied Sciences, Elverum, Norway; 4 Faculty of Health Studies, VID Specialized University, Sandnes, Norway; 5 Occupational Therapist, Rapides Parish School District, 619 6th St, Alexandria, LA 71301 USA

**Keywords:** Education, Graduate; learning, Occupational therapy, Physical therapy, Mental health, Self-efficacy

## Abstract

**Background:**

Occupational and physical therapy academic programs are rigorous. Increased rates of student anxiety and depression may impact learning. Data on student study skills, self-efficacy, and mental health is limited. This study explored relationships between students’ self-efficacy, mental health factors, and approaches to studying.

**Method:**

A cross-sectional study was designed. Seventy-three students completed the Approaches and Study Skills Inventory for Students-Short Form, General Self-Efficacy Scale, and Mental Health Continuum-Short Form. Associations between predictors (education program, general self-efficacy and mental health) and ratings on the study approach scales were analyzed with multiple linear regression.

**Results:**

Multiple regression models revealed associations between higher self-efficacy and higher ratings on the deep (β = 0.49, *p* <  0.01) and strategic (β = 0.34, *p* <  0.05) scales, and lower ratings on the surface scale (β = − 0.29, p <  0.01). Compared to OT students, PT students had higher surface approach ratings (β = − 0.36, *p* <  0.001). Poorer mental health scores were associated with higher surface approach ratings (β = − 0.41, *p* < 0.001).

**Conclusions:**

To support productive study strategies among occupational and physical therapy students it may be useful to promote their general self-efficacy and positive mental health.

## Background

There is an increasing demand for occupational therapy (OT) and physical therapy (PT) services [1] suggesting a need for increased enrollment in these educational programs. OT and PT programs have rigorous curricula in diverse areas such as physical and social sciences, research methods, psychopathology and interviewing skills; their accrediting bodies have established extensive guidelines for curricula that include goals of creating adaptable, competent, and self-assured practitioners [2, 3]. Additionally, graduates must demonstrate professional competence by passing school and national exams before they can practice. Complicating this is the growing concern about student mental health and sense of competence to manage academic demands. Evidence suggests that negative aspects of student mental health, such as anxiety, depression and distress have increased [4, 5]. These apparent increases in negative mental health, and limited data pertaining to graduate student competence and self-efficacy, create a compelling need to explore these factors and potential associations with approaches to studying.

Approaches to studying are strategies individuals employ to gain, process and use new knowledge. Marton and Säljö [6] identified two ways students process information during learning: the deep and surface approaches to learning. Students who utilize a deep approach actively engage with material to internalize the fundamental meaning of information presented, while those using a surface approach use memorization and rote learning to passively acquire information presented [7]. Some years later, a “strategic approach” was added to characterize students who focus on organizational aspects of learning and actively monitor and revise their use of time and effort to be academically successful [7].

Several environmental factors influence students’ choice of study approaches. Learning context and student perceptions of tasks have been identified as having significant influences on how students approach studying [8]. As examples, the perception of “good teaching,” freedom in learning, and adequate interest level in subjects appear to be primary factors in choosing deep approaches [8, 9]. Surface approaches are connected to perception of heavy workload, lack of freedom in learning, and extrinsic motivations such as a fear of failure [8, 9]. Having a holistic understanding of student approaches to learning and factors that impact their choices may help educators facilitate environments that promote learning and result in graduates who are better prepared for practice.

Recent research reveals connections between approaches to studying and academic performance. Correlations were found between positive student outcomes and adoption of deep and strategic approaches for undergraduate OT students [10]. However, research into OT students’ study approaches has been primarily conducted with undergraduates. Undergraduate student research is an important start, however there is a clear lack of research on graduate OT and PT students’ approaches to studying.

Self-efficacy is understood as the inherent belief that an individual possesses the skills needed to accomplish a desired task or goal [11]. Self-efficacy reflects a person’s self-confidence in how they think and are motivated to approach activities [11]. In education, self-efficacy refers to a student’s belief that he or she can perform academic tasks [12]. Therefore, higher self-efficacy is likely to be related to academic success. Bonsaksen’s [13] study of undergraduate OT students showed higher self-efficacy was associated with higher levels of program satisfaction. Additionally, van Lankveld et al. [14] found levels of self-efficacy were strong predictors of PT students’ success with work and study in specific skill areas. Other studies found connections between self-efficacy and academic task performance in the specific skill areas of therapeutic use of self [15], wheelchair skills training [16], acute care setting work skills [17], and interviewing skills [18]. No studies on relationships between general self-efficacy and academic performance of graduate OT and PT students were found. The World Health Organization (WHO) defines mental health as comprising self-realization, adequate coping skills, and the ability to engage in activities that contribute to one’s community [19]. Thus, mental health is not just the absence of illness but encompasses wellbeing and productive individual and community functioning. Keyes [20] defined positive mental health as positive appraisals of emotional (EWB), psychological wellbeing (PWB), and social wellbeing (SWB). Undergraduate students high in both EWB and SWB factors have greater academic performance, with EWB factors of personal growth/purpose and motivation being the strongest predictors of academic success [21]. And, students who are motivated by intrinsic desires to explore, learn new concepts, and derive pleasure from the process report an increased sense of well-being and life satisfaction, and demonstrate better academic performance [22]. Thus, increased experiences of positive mental health factors may enhance academic performance. The relationships between how a student approaches studying and their sense of self-efficacy and positive mental health factors are complex. Both self-efficacy and positive mental health factors have been linked to intrinsic motivation, which has been shown to impact the ways students approach studying ([10, 23]. Conversely, lower reported use of deep learning approaches correlated to low self-efficacy, showing that changes in study approaches are related to self-efficacy beliefs [23].

While some research links student approaches to studying with positive mental health factors, the literature more often focuses on the impact of *negative* mental health factors. For example, Cipra and Müller-Hilke [24] found positive correlations between students who employ a surface approaches and high levels of reported anxiety, and relationships between lower reported anxiety and students who utilize strategic approaches to studying. While this may indirectly speak to positive mental health factors, this was not the intention of the study. A review of available literature indicates that the impact of positive mental health factors on studying approaches remains largely unexplored in OT and PT graduate students.

The purpose of this study was to explore graduate OT and PT student approaches to studying, self-efficacy and positive mental health factors and the relationships between them. This study’s overall research question was: How do general self-efficacy and positive mental health relate to approaches to studying in OT and PT doctoral students?

## Methods

### Study design and commencement

This cross-sectional survey design study was conducted in the fall of 2019.

### Participants and recruitment

The authors confirm that all methods were carried out in accordance with relevant guidelines and regulations. Informed consent to participate was obtained after IRB committee approval. Participants were recruited from first-year students enrolled in the doctoral OT and PT programs via personal invitation and presentation of the study by ED and SM, in a California health sciences university. Students were encouraged to ask questions about the study and procedures, and were assured of their anonymous participation. All students had bachelor’s degrees or higher upon entry and responded between weeks three and five of their graduate education.

### Data collection

The Approaches and Study Skills Inventory for Students short version (ASSIST) is an 18-item self-report questionnaire that identifies student approaches to learning and studying in higher education and measures student engagement in deep, surface, and strategic learning approaches [25]. Participants rate the degree to which they agree or disagree with statements on a scale from 1 (disagree) to 5 (agree). Six items pinpoint student preference for each approach: surface, deep, and strategic. In this study, a preliminary factor analysis confirmed construct validity of the ASSIST scales by showing that all of the 18 items loaded on three factors exactly as expected from theory. Internal consistency (Cronbach’s α) was 0.62 (mean inter-item correlation 0.22) for the deep approach scale, 0.73 (mean inter-item correlation 0.33) for the strategic approach scale, and 0.80 (mean inter-item correlation 0.40) for the surface approach scale.

The General Self-Efficacy Scale (GSE) is a tool commonly used for operationalizing the concept of self-efficacy. The GSE is a 10-item questionnaire for measuring general self-efficacy with scores positively correlating with optimism and work satisfaction and negatively correlated with depressive and anxious thoughts, stress, and burnout [26]. Each GSE item includes a statement that indicates general self-efficacy, to which participants respond on a 4-point Likert scale, 1 (not at all true), to 4 (exactly true). Scoring ranges from a minimum score of 10 to a maximum score of 40, with higher scores indicating higher levels of self-efficacy. Internal reliability for the GSE was determined by Cronbach’s α, which was found to lie between 0.76 and 0.90 (Schwarzer and Jerusalem, 1995). In the current study’s preliminary factor analysis, however, item two (i.e. “If someone opposes me, I can find the means and ways to get what I want”) showed a communality of 0.04, indicating that it contributed very little to the variance in the latent factor, and its loading on the factor (0.20) was below the commonly applied threshold of 0.40. Thus, item 2 was removed from the scale, after which all items loaded substantially (0.50–0.70) on one latent factor. Scores on the resulting nine-item total scale were adjusted to account for the removal of item 2. Internal consistency (Cronbach’s α) for the revised GSE scale was 0.80 (mean inter-item correlation = 0.31).

The Mental Health Continuum-Short Form (MHC-SF) questionnaire is a 14-item tool used to identify positive mental health factors by focusing on emotional, psychological, and social well-being [27]. EWB items measure constructs such as happiness, interest in life, and life satisfaction. SWB items assess perceptions of social, social integration, social actualization, social acceptance, and social coherence. PWB items measure the constructs of self-acceptance, environmental mastery, positive relations with others, personal growth, autonomy, and purpose in life. Items are rated on a 6-point Likert scale that ranges from: 1 (never) to 6 (every day). Respondents are categorized as having flourishing, moderate, or languishing mental health based on reported experiences [27]. The MHC-SF has excellent levels of internal consistency (> 0.80) and validity based on past data obtained from adolescents and adults [28]. In this current study, all of the 14 items loaded substantially (0.49–0.88) on the one latent factor, indicating good construct validity. Internal consistency (Cronbach’s α) for the MHC-SF scale was 0.94 (mean inter-item correlation = 0.52).

### Statistical procedures

Data analysis was completed using SPSS® version 26. Pearson *r* correlation analyses were used to determine the unadjusted relationships between independent variables (positive mental health and self-efficacy) and the dependent variables (approaches to studying). Guidelines used for interpreting correlation coefficients were: (a) 0.00 to 0.25 for little or no relationship; (b) 0.25 to 0.50 for weak relationships; (c) 0.50 to 0.75 for moderate to good relationships; (d) above 0.75 for excellent relationships between two variables [29]. Differences in proportions between groups were examined with Chi-Square tests, and group differences on continuous measures were investigated with independent t-tests. Linear regression analyses were used to examine adjusted associations between general self-efficacy and mental health, and approaches to studying. Analyses were adjusted by affiliation to OT/PT program. Adjustment by age and gender were deemed unnecessary, as the unadjusted associations between age, gender and each of the study approaches were below r = 0.10 and not statistically significant. Coefficients of determination (explained variance; r^2^) were used to determine accuracy of predictions made using a regression analysis.

## Results

### Sample characteristics

Out of 86 eligible participants, 77 doctoral students (response rate of 89.5%) completed the study. Four of the responses were incomplete and thus removed;. Thus thus, the final sample consisted of 73 students in total; 35 from OT and 38 from PT. The participating students are described in Table 1. More than half of both groups were comprised of women however, compared to PT students (55.3%), females represented the majority of OT students (88.6%, *p* < 0.01). PT students had significantly higher scores on the ASSIST’s surface approach scale (M = 18.2, SD = 4.7), compared to OT students (M = 14.9, SD = 4.9, p < 0.01). Otherwise there were no significant differences between the groups on the employed measures therefore the groups were combined as one in subsequent analyses.

**Table 1:** Characteristics of the study sample (*n* = 73)

Characteristics	Total (n = 73)	OT students (*n* = 35)	PT students (*n* = 38)	p	Cohen’s d
Age	n (%)	n (%)	n (%)		
Below 30 years	53 (72.6)	27 (77.1)	26 (68.4)	0.40	
30 years or above	20 (27.4)	8 (22.9)	12 (31.6)		
Sex	n (%)	n (%)	n (%)		
Male	21 (28.8)	4 (11.4)	17 (44.7)	< 0.01	
Female	52 (71.2)	31 (89.6)	21 (55.3)		
	M (SD)	M (SD)	M (SD)		
General self-efficacy	31.7 (3.5)	31.8 (4.1)	31.6 (2.9)	0.87	0.06
Mental health (total)	44.8 (13.7)	43.3 (14.6)	46.2 (12.9)	0.38	−0.21
Emotional well-being	10.5 (3.5)	10.4 (3.4)	10.6 (3.6)	0.78	−0.06
Social well-being	13.7 (5.8)	12.8 (6.3)	14.4 (5.3)	0.25	−0.27
Psychological well-being	20.6 (5.9)	20.1 (6.4)	21.1 (5.5)	0.47	−0.19
Study approaches	M (SD)	M (SD)	M (SD)		
Deep approach	22.2 (3.8)	22.4 (3.9)	22.0 (3.7)	0.70	0.11
Strategic approach	24.5 (4.0)	24.3 (4.7)	24.6 (3.2)	0.74	−0.07
Surface approach	16.6 (5.1)	14.9 (4.9)	18.2 (4.7)	< 0.01	−0.69

Note. Statistical tests are Chi-square test (for age groups and sex) and independent samples t-test (for all other variables)

### Bivariate correlations

Responses to the MHC-SF showed moderate, positive correlations with the GSE scale (*r* = 0.60 *p* < 0.01), showing participants with greater positive mental health factors reported higher levels of self-efficacy. The MHC-SF and the ASSIST’s surface approach to studying had a moderate, negative correlation (*r* = − 0.54, *p* < 0.01), showing participants with greater positive mental health factors used fewer surface studying behaviors. The MHC-SF and the ASSIST’s strategic approach to studying demonstrated a weak, positive correlation (*r* = 0.40, *p* < 0.01), indicating participants with greater positive mental health factors were more likely to use the strategic approach to studying.

The GSE scale and the ASSIST’s surface approach to studying revealed a moderate, negative correlation (*r* = − 0.54, *p* < 0.01), indicating participants with higher reported self-efficacy were less likely to use surface studying behaviors. The strategic approach demonstrated a weak, positive correlation with the GSE (*r* = 0.46, *p* < 0.01), indicating participants who reported higher levels of self-efficacy were more likely to use strategic approaches. The ASSIST’s deep approach had a weak, positive correlation with the GSE (*r* = 0.39, p < 0.01), showing participants who reported higher levels of self-efficacy were more likely to use deep study approaches.

### Adjusted associations with study approaches

Regression analyses are displayed in Table 2. Higher ratings on general self-efficacy were associated with higher deep approach ratings (β = 0.49, *p* < 0.01), and the full model explained 17.3% of the variance in deep approach ratings. Similarly, higher ratings on general self-efficacy were associated with higher strategic approach ratings (β = 0.34, *p* < 0.05), and the full model explained 23.2% of the variance in strategic approach ratings. Compared to OT students, the PT students had higher surface approach ratings (β = − 0.36, *p* < 0.001), even when adjusting by self-efficacy and positive mental health. Higher ratings on general self-efficacy (β = − 0.29, p < 0.001) and positive mental health (β = − 0.41, p < 0.001) were associated with lower surface approach ratings.

**Table 2:** Associations with deep, strategic and surface approach scale ratings (n = 73)

	Deep approach			Strategic approach			Surface approach		
Independent variables	b	95% CI	β	b	95% CI	β	b	95% CI	β
Education program	0.14	−1.49 – 1.77	0.02	−0.22	−1.87 – 1.43	−0.03	−3.61	−5.32 - -1.91	− 0.36***
General self-efficacy	0.53	0.24–0.82	0.49**	0.39	0.10–0.68	0.34*	−0.42	− 0.72 - -0.12	−0.29**
Mental health	−0.05	−0.12 – 0.02	− 0.17	0.06	− 0.01 – 0.13	0.19	− 0.15	−0.23 – 0.07	− 0.41***
Explained variance		17.3%**			23.2%***			49.4%***	

Note. Explained variance indicates the variance proportions of the dependent variable accounted for by the independent variables together. Education program coding: Physiotherapy = 0, occupational therapy = 1. 95% confidence intervals (CI) are constructed by adding and subtracting the standard error (SE) multiplied by 1.96 to/from the unstandardized estimate (b)

***p < 0.001

**p < 0.01

*p < 0.05

## Discussion

This study explored how general self-efficacy and positive mental health are related to approaches to studying in doctoral-level OT and PT students. Higher levels of general self-efficacy were associated with higher ratings on deep and strategic study approach scales, and with lower ratings on the surface approach scale in this study. Of the combined group of students, those with lower positive mental health ratings were more inclined to use the surface approach to studying. PT students overall, and OT and PT students with lower mental health ratings were more inclined to employ the surface approach to studying.

This study demonstrated an association between lower positive mental health and the use of more surface approaches. Many students also reported using both deep and surface approaches to studying. The use of contrasting study behaviors seems to resonate with others’ findings, such as Prat-Sala and Redford [23] who found a similar combined use of seemingly opposite deep and surface approaches in their large study of undergraduate students representing a variety of disciplines. The authors hypothesized that students likely used these in a complimentary fashion, based on perceived demand of the content, thus making it context-dependent [23]. However, it could be students with poorer mental health feel more secure if they try to absorb all course information, which may be attainable only via rote memorization, a surface approach to learning [7]. An emphasis on rote memorization may likewise result in lost opportunities for making deeper connections between different concepts and ideas that are taught in the course. Thus, striving to succeed via rote memorization may be counterproductive if assessments of learning are oriented towards the students’ application of broad concepts.

Other studies show student approaches to studying are likely influenced by external factors. For example, surface approaches are more often associated with students in the “hard” sciences, such as biology or chemistry, whereas those in the “soft” (social and behavioral sciences) favor deeper approaches, such as relating knowledge to other areas of study [27, 30]. It is possible that the PT curriculum in this study is more strongly oriented to the hard sciences than the OT curriculum, which could account for PT students’ use of more surface approach behaviors. Learning and teaching may also be influenced by professional paradigms that could be instilled in students as they progress through professional programs. For example, OT education in the United States has been guided by the concepts of active learning [31], which are associated with the deep approach to learning [7]. This is particularly true in the current study program. Although active learning may be considered more effective for student learning [32], other findings indicate that successful active learning is critically dependent on the facilitators’ skill at guidance, given in accordance with the subject matter and learner characteristics [33]. Exploration of the differences of “hard” versus “soft” sciences, and the use and effectiveness of active learning in the OT and PT curricula may shed light on this.

The reported high sense of self-efficacy in this study’s participants’ is considered a positive finding that supports other studies of OT student samples, such as Bonsaksen’s 2015 [13] study revealing similarly high levels of self-efficacy. Likewise, a study assessing self-efficacy among PT students showed a mean GSE score of 3.1 per item, compared to the present study’s student average of 3.2 per GSE item [14]. These similarities further support analysis of the two groups’ data as one. GSE mean scores in the current study were also consistent between the genders, a somewhat unexpected finding, since other studies have found that male OT and male medical students report higher self-efficacy than their female counterparts [13, 34].

Although the students’ scores indicate they have overall positive self-efficacy, situations with higher social demands, such as completing group projects or giving one another feedback may evoke greater stress and may be reflected in the results. In fact, Curran and Hill [35] note that socially prescribed perfectionism (or perceived demands from others to behave and perform flawlessly) has risen dramatically in college students. Thus, there is potential for these socially referenced stressors to result in poor coping for students, since some corrective feedback may be given in front of others [35]. Likewise, it seems possible that assignments that require interactive, social processes may exacerbate this. Nonetheless, the collective overall higher sense of self-efficacy in this group is interpreted as positive, since research shows self-efficacy is associated with higher academic success and higher self-regulation in learning [36].

The GSE assesses general self-efficacy in a broad sense. However, it may be more effective to gauge self-efficacy in specific areas as there appears to be a direct relationship between skill-specific self-efficacy and skill performance as described by Schutte and Malouff [37], which is highly relevant to clinical professions. Pasupathy and Bogschutz [38] found that speech-language pathology students that reported higher levels of clinical self-efficacy were judged to have higher clinical performance skills by faculty. Both general and specific self-efficacy have been associated with increased competency and function in professional roles and appear to have a bidirectional relationship, which has clear importance to educators teaching future clinical providers [37]. These results suggest that students who gain competence in specific clinical skills could develop a higher overall sense of self-efficacy. Moreover, higher levels of specific self-efficacy can lead to increased self-efficacy in other professional areas such as leadership [39] a skill area described as essential to OT and PT practice [2, 3, 40].

The majority of participants in this study reported experiencing positive mental health. This is considered a positive finding, as prior research shows positive mental health is associated with higher levels of academic success [21]. While participants reported positive mental health factors, graduate school often presents novel academic, cognitive, and self-management challenges for students [41]. Results of this study may reflect the impact of these challenges and could indicate risk, but also represent potential areas for interventions to enhance student positive mental health and engagement. For example, civic engagement has been associated with positive mental health factors for college students [42] and may be one such area for intervention. This concept also resonates with foundations of OT practice, to enhance individual, societal and global wellbeing for all humans [3]. One model of civic engagement in allied health educational programs, service learning, is already being incorporated with positive results, which may likewise be reflected in our findings [43]. Another recent study of a multi-faceted self-care intervention for graduate students showed promising results in increasing self-care habits that correlated with decreased levels of psychological stress, depression, and anxiety, and increases in social life satisfaction [44]. These represent potential intervention areas to enhance student mental health and sense of self-efficacy, potentially impacting their overall approaches to studying using concepts that clearly reflect tenets of OT practice [3].

### Limitations and future directions

This study explored only 73 participants’ experiences in a small university in the western USA. No academic information was yet available to the researchers since the protocols were given during the first month of school. Thus, no conclusions can be drawn about how the particular constellations of general self-efficacy, positive mental health and studying/learning approaches might impact academic performance in this group. Future research could look at changes in all of the factors over time and compare these to academic outcomes. These data would have potential importance to curriculum development, faculty development opportunities, and areas of targeted student support to enhance academic self-efficacy, and positive mental health outcomes.

## Conclusion

This is a preliminary study of student approaches to studying, self-efficacy, and positive mental health, with opportunities for future participant follow up and further analysis. The constructs of mental health, self-efficacy, and approaches to studying seem to be interconnected. Each construct may impact the other and have a cascade of effects on student success. Therefore, this research reveals opportunities for potential academic/teaching-based interventions and student-based interventions that could positively impact student outcomes. Specifically, to support productive study strategies among students in OT and PT, it may be useful to promote their general self-efficacy and positive mental health.

## Data Availability

The datasets used and/or analyzed during the current study are available from the corresponding author on reasonable request.
